# Impact of Testosterone on Male Health: A Systematic Review

**DOI:** 10.7759/cureus.82917

**Published:** 2025-04-24

**Authors:** Julio G Rojas-Zambrano, Augusto Rojas-Zambrano, Andres F Rojas-Zambrano

**Affiliations:** 1 Gynecology, Dr. Regeneración, Guayaquil, ECU; 2 General Medicine, Dr. Regeneración, Guayaquil, ECU; 3 Medicine, Dr. Regeneración, Guayaquil, ECU

**Keywords:** bone strength, male hormone, men, mood, muscle mass, testosterone

## Abstract

The development and maintenance of muscular mass, bone density, and general physical strength depend heavily on testosterone, a hormone that plays a complicated and significant role in men's physiology. Beyond these physical advantages, testosterone plays a crucial role in male reproductive health by affecting spermatogenesis (the generation of sperm), libido, and erectile function. It has an effect on many body systems, underscoring its importance for men's physical health and fertility. The goal of this study is to learn more about the critical and varied roles that testosterone plays in healthy men's physiological functioning. The following MeSH terms were used in a PubMed search that covered the years 1998 to the present: (((testosterone) OR (androgens)) OR (testosterone insufficiency)) AND (healthy men)) AND (testosterone replacement). Descriptive, observational, and experimental studies including healthy men-more especially, those assessing the effects of testosterone therapy-were required for inclusion. Testosterone supplements can have a good impact on a number of important aspects of men's health, such as vascular endothelial function, mood (particularly in lowering depression), muscle strength, bone health, and sexual function. Small sample sizes and a dearth of studies, however, limit these findings, highlighting the need for more investigation to completely comprehend the wider impacts of testosterone on men's health. Testosterone therapy has modest advantages, especially for men who have hypogonadism symptoms and low testosterone levels. These advantages include better vascular function, mood, muscle strength, bone density, and sexual health in healthy men. Considering the prospective benefits of testosterone therapy, more investigation and clinical testing are necessary to completely comprehend its effects and improve therapeutic modalities. Further research will better define the function of testosterone in both healthy and deficient men, which will eventually result in more accurate and successful treatment plans.

## Introduction and background

A key component of human physiology is hormonal regulation, which intricately regulates a wide range of biological processes and maintains homeostasis across multiple systems; from the many hormones that regulate bodily functions, testosterone stands out for its significant and complex effects on male health [1]. An outline of the major hormonal changes that take place in men throughout their lives is given in this introduction, with special attention to testosterone's crucial role in controlling a wide range of physiological processes. The objective of this systematic review is to critically analyze the various functions of testosterone, including its physiological importance, regulatory processes, and possible repercussions if it is dysregulated in men.

Human development involves hormonal changes that start in fetal life and last into adulthood. In men, the endocrine system, which includes glands like the pituitary, thyroid, adrenal, and gonads, releases hormones that control important functions like growth, metabolism, reproduction, and mood [2]. In addition to having significant effects on health and illness, these hormonal changes are necessary for preserving physiological balance; one particularly significant time of hormonal change is during puberty, when a rise in sex hormones, such as testosterone, promotes the growth of secondary sexual traits like increased muscle mass, body and facial hair, and a deeper voice [3].

Testosterone, a steroid hormone predominantly synthesized in the testes, is integral to a wide range of physiological processes that are crucial to male health; the regulation of testosterone levels operates through a feedback mechanism that is essential for understanding its physiological control. Specifically, when testosterone levels rise, the body reduces the production of its primary source, while a decline in testosterone levels prompts an increase in its production; this intricate feedback loop plays a critical role in maintaining hormonal balance and is essential for understanding the regulatory mechanisms governing testosterone in men [1]. Dysregulation of this feedback mechanism can lead to a variety of pathophysiological conditions, particularly testosterone deficiency; such disruptions may occur due to aging, disease processes, or lifestyle factors, underscoring the importance of accurate diagnosis and appropriate therapeutic interventions [4].

The goal of this systematic review is to provide a thorough examination of testosterone's function in relation to the larger framework of hormonal changes that occur during the male lifecycle; this review attempts to give a better knowledge of testosterone's crucial function in male health and disease by pointing out gaps in the existing data and considering possible directions for further study. Additionally, it aims to clarify the effects of testosterone dysregulation on men's mental and physical wellness while advancing clinical procedures and treatment approaches.

## Review

The synthesis and regulation of testosterone, a vital steroid hormone, are highly complicated procedures that involve intricate interactions among multiple endocrine glands, including the hypothalamus, pituitary gland, and gonads, as well as feedback mechanisms that maintain homeostasis within the body. In men, testosterone is primarily synthesized in the testes and the adrenal gland, with smaller amounts produced in the ovaries and adrenal glands in women [2].

The Leydig cells of the testes are the main source of testosterone in men; although testosterone is frequently regarded as a male hormone, it is essential for many physiological processes outside of sexual differentiation, including maintaining muscle mass, bone density, mood regulation, and metabolic processes [1]. The hypothalamic-pituitary-gonadal (HPG) axis, a crucial part of the endocrine system, controls the production of testosterone. It involves dynamic interactions between the gonads, pituitary gland, and hypothalamus. The brain's hypothalamus starts the hormonal cascade by secreting gonadotropin-releasing hormone (GnRH), which causes the pituitary gland to release follicle-stimulating hormone (FSH) and luteinizing hormone (LH) [1].

The production of testosterone in men is primarily controlled by negative feedback mechanisms, whereby high levels of testosterone prevent the release of GnRH from the hypothalamus and LH from the pituitary, thereby limiting further testosterone synthesis; testosterone is made from cholesterol by a variety of enzymatic pathways in the testes [2].

The enzyme 5α-reductase transforms testosterone into dihydrotestosterone (DHT). DHT has a stronger androgenic effect and is essential for the maintenance of prostate health and the development of secondary sexual traits in men [2]. The enzyme aromatase in men can aromatize testosterone into estrogen, which affects a variety of physiological processes, such as bone health and reproductive capabilities; the average testosterone level in healthy men is between 264 and 916 ng/dL [5].

Libido, or sexual desire, is significantly influenced by testosterone, which regulates various brain regions involved in sexual motivation, including the hypothalamus; in men, testosterone plays a crucial role in sexual desire and arousal [6]. Lower testosterone levels are often associated with decreased libido in men, leading to reduced sexual activity and satisfaction; testosterone therapy may help restore libido and improve sexual function in men with low testosterone levels [3].

As a primary hormone influencing bone metabolism, testosterone directly affects osteoclasts, osteoblasts, and osteocytes, promoting periosteal bone formation during puberty and decreasing bone resorption during adulthood; testosterone is also strongly correlated with bone density; lower testosterone levels result in decreased bone density [7].

The increase in sex steroid production during puberty speeds up bone mineral accumulation and causes sex-specific variations in bone growth; after mid-puberty, the male population experiences a greater increase in periosteal bone growth than the female population, who shows more pronounced endocortical bone formation [4]. Essential for bone maturation, testosterone helps bones reach maximal mass and preserves bone density, all during adulthood; it also promotes skeletal growth by improving mechanical loading [8].

Because it promotes protein synthesis and muscle hypertrophy through androgen receptor binding in muscle cells, testosterone is a critical regulator of muscle mass; this anabolic impact is required for muscle development and repair, making testosterone crucial for preserving muscle mass [9]. Because testosterone influences neuromuscular function, which includes muscle coordination and force output, it has a substantial impact on muscle strength; higher testosterone levels are associated with better physical performance and muscle strength [10].

It has been demonstrated that testosterone replacement treatment (TRT) improves muscle mass, strength, and physical function in hypogonadal men, while research indicates that men with low testosterone levels have decreased muscle mass and strength [10]. The likelihood of sarcopenia (age-related muscle loss) and diminished muscle strength rises as testosterone levels naturally fall with age; however, research indicates that testosterone therapy may mitigate these effects in older men, enhancing physical performance, sexual drive, and muscle mass [11].

Testosterone also has a substantial impact on mental health; depression symptoms are associated with low testosterone levels, and testosterone therapy has the potential to help some people with their depressive symptoms [12]. In men with hypogonadism, including elderly individuals, testosterone replacement therapy may offer antidepressant effects, providing therapeutic benefits for those with testosterone deficiency [13].

Additionally, testosterone supports endothelial function by increasing nitric oxide production, encouraging endothelial cell growth and repair, and lowering inflammation; it also has a significant impact on the vascular endothelium, the thin layer of cells lining blood vessels, which is essential for preserving cardiovascular health [14].

Materials and methods

**Figure 1:**
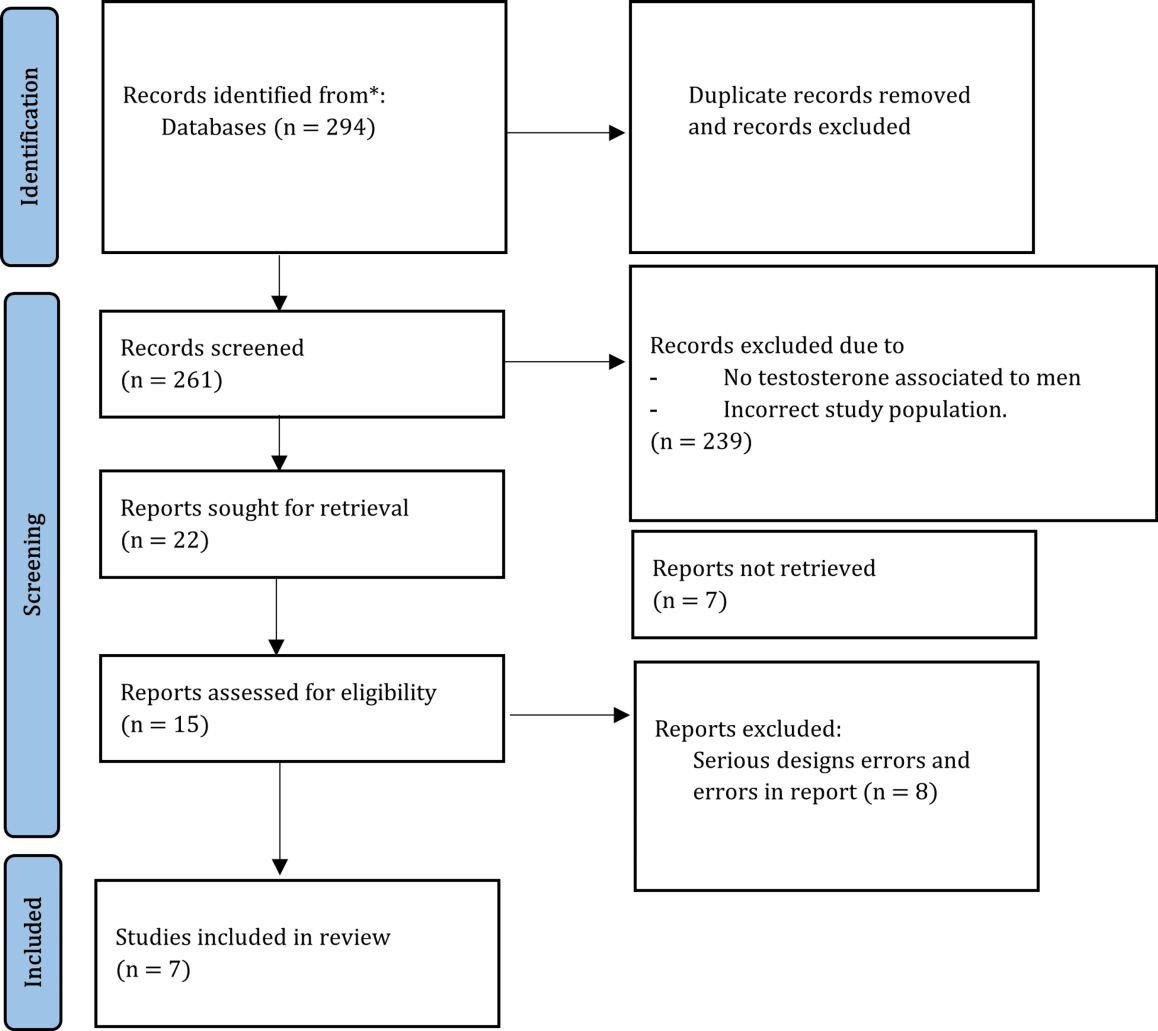
PRISMA method PRISMA: Preferred Reporting Items for Systematic Reviews and Meta-Analyses

Following the recommendations outlined in the Preferred Reporting Items for Systematic Reviews and Meta-Analyses (PRISMA) statement, we conducted a literature search [15]. It is significant to emphasize that since this study reviews already published studies pertaining to patient data, ethical approval is not necessary. The following database was thoroughly searched: ((testosterone) OR (androgens)) OR (testosterone insufficiency)) AND (healthy patients)) AND (testosterone replacement)) is the MeSH term that was applied by PubMed. From 1998 to the current piece, we used data. Based on the predetermined criteria, publications that evaluated the effects of testosterone using descriptive, observational, or experimental designs involving human subjects were included. The research paper provides a thorough summary of the selection criteria and covers testosterone's effects on depression, vascular endothelium, muscle strength, bone health, and sexual function (Figure 1). We provide a table that summarizes all the data gathered on the comparison of different factors linked to the ones previously discussed in order to improve comprehension of the data.

Results

As shown in Figure 1, our investigation started with an initial collection of 294 articles. Thirty-three papers were eliminated for duplication after an initial examination of the abstracts and titles. A further 239 records were subsequently removed during the full-text screening stage; seven articles were, thus, left for an in-depth examination.

Seven studies were eventually included in the systematic review (Table 1), and all of them showed beneficial impacts on a range of health outcomes, including depression, vascular endothelial function, muscle strength, bone health, and sexual function. These results demonstrate the possible advantages in these fields. However, the use of a carefully planned randomized controlled trial (RCT) is advised in order to bolster the evidence even further and offer more conclusive findings.

**Table 1:** Descriptive analysis

Author(s), years	Study design	Aim of study	Setting and participants	Results and findings
Mlynarz et al. (2024) [16]	Systematic review	This study seeks to assess the direct impact of testosterone therapy on the components of metabolic syndrome, while excluding individuals with type 2 diabetes [16].	2 meta-analyses	This study indicates that testosterone therapy leads to improvements in the components of metabolic syndrome [16].
Cruickshank et al. (2024) [17]	Systematic review with meta-analysis	The primary objective was to evaluate the safety of testosterone replacement therapy [17].	35 trials	Testosterone replacement therapy improves sexual function and quality of life, without adversely affecting blood pressure, serum lipids, or glycemic markers [17].
Xu et al. (2024) [18]	Systematic review with meta-analysis	Evaluate the impact of testosterone replacement therapy on erectile function and prostate health [18].	This analysis reviewed 28 randomized controlled trials (RCTs) involving a total of 3,461 patients	This meta-analysis of RCTs indicates that testosterone replacement therapy (TRT) may improve erectile function in men with hypogonadism [18].
Yang et al. (2023) [19]	Systematic review and meta-analysis	The aim was to conduct a systematic review and meta-analysis to determine whether testosterone replacement therapy (TRT) can enhance sexual function in the elderly [19].	5 randomized controlled trials (RCTs)	The improvement in both erectile function and sexual motivation was clearly apparent [19].
Mauvais-Jarvis and Lindsey (2024) [20]	Systematic review	This review explores the metabolic benefits of estradiol and testosterone in both sexes [20].	157 references	Testosterone plays a key role in promoting bone formation [20].
Vartolomei et al. (2020) [21]	Systematic review	The objective is to investigate and critically assess the existing evidence on the effects of testosterone replacement therapy (TRT) on depression and depressive symptoms in adult men with late-onset testosterone deficiency, compared to a placebo [21].	1,586 participants	Data from small, placebo-controlled randomized clinical trials involving patients with mild depression before treatment suggest that testosterone replacement therapy (TRT) is linked to a reduction in depressive symptoms [21].
Kelly and Jones (2013) [22]	Narrative review	The aim is to assess the role of the vascular hormone in both health and disease [22].	Review of 8 articles	Testosterone has demonstrated clinically significant anti-inflammatory effects, and testosterone replacement therapy (TRT) has been shown to improve atherosclerosis, as assessed non-invasively in hypogonadal men and in animal studies [22].

A higher level of evidence would be provided by a well-conducted RCT, improving the consistency and dependability of the results and providing a more thorough knowledge of the effects seen across studies, so we could find more evidence in the future.

Each study's risk of bias was evaluated, as shown in Table 2. Potential biases were assessed by two independent reviewers (JR and AR) in four crucial areas: reporting bias, confounding factors, measurement bias, and selection bias. A "low," "moderate," or "high" risk of bias was assigned to each domain. The selection, screening, and assessment of the chosen studies were assisted by the remaining authors, AR and AFR. The majority of research was determined to have a moderate likelihood of bias.

**Table 2:** Risk of bias assessment

Study	Selection bias	Measurement bias	Confounding	Reporting bias	Overall risk of bias
Mlynarz et al. (2024) [16]	Low	Moderate	Low	Low	Low
Cruickshank et al. (2024) [17]	Moderate	Low	Moderate	Moderate	Moderate
Xu et al. (2024) [18]	Moderate	Low	Low	Low	Low
Yang et al. (2023) [19]	Moderate	Low	Moderate	Moderate	Moderate
Mauvais-Jarvis and Lindsey (2024) [20]	Low	Moderate	Low	Low	Low
Vartolomei et al. (2020) [21]	Low	Low	Moderate	Moderate	Low-moderate
Kelly and Jones (2013) [22]	Low	Low	Moderate	Moderate	Low-moderate

Discussion

Testosterone plays a crucial role in regulating libido in both men and women by influencing brain regions associated with sexual motivation and arousal [17]. Another research points out that a low testosterone level is associated with low libido [23]. Additionally, there is a significant correlation between bone density and testosterone levels; a decrease in testosterone can result in decreased bone density [20]. It is also critical to comprehend how the Free Androgen Index (FAI), which is connected with bone density, is used; a low FAI indicates a higher likelihood of bone density loss [24].

Furthermore, via binding to androgen receptors in muscle cells, testosterone stimulates protein synthesis and muscular growth, which is crucial for the regulation of muscle mass [10]. Our understanding of the relative advantages of both physiological and pharmacological therapies for aging men is greatly improved by the effects of testosterone treatment and exercise on factors such as strength, aerobic fitness, and body composition [25].

Regarding mental health, testosterone may help certain people with their depression symptoms; this is especially important for patients with hypogonadism, such as elderly people, for whom testosterone replacement therapy may be quite beneficial [13]. Moreover, by encouraging the synthesis of nitric oxide, aiding in the development and repair of endothelial cells, and lowering inflammation, testosterone improves endothelial function, which is essential for preserving vascular health and underscoring testosterone's complex role in general physiological well-being [22].

Testosterone, like many medications, can have side effects, one of which is hirsutism-excessive growth of coarse, dark hair in areas typically associated with male-pattern hair growth; this happens in areas like the face, chest, and belly because testosterone stimulates the growth of new hair by binding to androgen receptors in hair follicles; those with a genetic predisposition to hyperandrogenism are more likely to experience more pronounced hair growth [26]. Therefore, to reduce this adverse effect, cautious monitoring is required.

Significant increases in hemoglobin and hematocrit levels have been linked to testosterone therapy; these physiological alterations may raise the risk of thromboembolic events; likewise, there is evidence that testosterone administration may cause a mild worsening of sleep-disordered breathing, especially in patients who have undiagnosed or underlying obstructive insomnia, as well as association with a possible decrease in high-density lipoprotein (HDL) cholesterol levels [25]. Currently, there are various approaches to treating patients with testosterone insufficiency, including the use of testosterone pellets and formulations combined with aromatase inhibitors, which need more studies for a better understanding of their effects [27]. 

In particular, the findings underscore the urgent need for well-designed RCTs in this area. By identifying limitations in existing studies and suggesting directions for future investigations, we hope to encourage the research community to pursue more robust and methodologically sound studies that will further strengthen the evidence base.

## Conclusions

Conclusively, testosterone is an essential hormone that is involved in many physiological processes throughout men's lives; in addition to controlling libido, testosterone is closely linked to bone density, muscle growth, and repair; its influence on mental health, especially in reducing symptoms of depression, emphasizes its complex nature. Additionally, testosterone plays a vital role in vascular health by improving endothelial function, which is crucial for maintaining cardiovascular health in men.

The physiological roles of testosterone in the male population are thoroughly examined in this systematic review, which also highlights the need for additional study to close current knowledge gaps; further investigation into the functions of testosterone may result in the creation of more potent treatment plans, which would ultimately enhance men's health and quality of life in a variety of demographics.
